# Enhanced ethanol sensing properties of WO_3_ modified TiO_2_ nanorods

**DOI:** 10.3906/kim-2008-46

**Published:** 2021-04-27

**Authors:** Bekzat ABDIKADYR, Alp KILIÇ, Onur ALEV, Serkan BÜYÜKKÖSE, Zafer Ziya ÖZTÜRK

**Affiliations:** 1 Department of Physics, Faculty of Science, Gebze Technical University, Kocaeli Turkey

**Keywords:** WO_3_/TiO_2_, gas sensor, heterostructure, nanorods, ethanol

## Abstract

Pristine and WO_3_ decorated TiO_2_ nanorods (NRs) were synthesised to investigate n-n-type heterojunction gas sensing properties. TiO_2_ NRs were fabricated via hydrothermal method on fluorine-doped tin oxide coated glass (FTO) substrates. Then, tungsten was sputtered on the TiO_2_ NRs and thermally oxidised to obtain WO_3_ nanoparticles. The heterostructure was characterised by X-ray diffraction (XRD), scanning electron microscopy (SEM), and energy-dispersive X-ray (EDX) spectroscopy. Fabricated sensor devices were exposed to VOCs such as toluene, xylene, acetone and ethanol, and humidity at different operation temperatures. Experimental results demonstrated that the heterostructure has better sensor response toward ethanol at 200 °C. Enhanced sensing properties are attributed to the heterojunction formation by decorating TiO_2_ NRs with WO_3_.

## 1. Introduction

Volatile organic compounds (VOCs) such as ethanol, acetone, toluene, xylene have been widely used in daily life, especially in industrial applications. However, VOCs induce some harmful effects for human health and environment [1-6]. In addition, some VOCs have been referred as tracer compounds in the human exhaled breath for various diseases [7-9]. Moreover, ethanol is the strongest indicator for detection of alcohol level in human breath [10]. Therefore, proper and fast detection of various VOCs is very important for different applications such as, traffic security, environmental monitoring, in-door air quality and breath analysis.

There are many detection techniques for VOCs such as optical, spectroscopic, chromatographic, electrochemical [11-13]. Among all these techniques, semiconductor metal oxide (SMO) based chemi-resistive gas sensors are one of the best candidates due to their high sensitivity and easy production processes [14-16]. Moreover, different techniques such as loading with a catalyst, doping a host element, or heterostructural fabrication of SMO materials may improve their gas sensing properties against various gas species [17-22]. Therefore, SMOs are superior sensing materials for VOCs detection.

As an n-type semiconductor, TiO_2_ is one of the most used material for VOCs detection for many years. TiO_2_ has some distinctive features such as its nontoxic nature, easy nanostructural fabrication, and superior reaction ability to a wide range of VOCs. Especially, one dimensional (1D) TiO_2_ nanomaterials such as nanorods and nanotubes have been widely used due to their higher surface-to-volume ratio [23-27]. However, some sensor properties such as selectivity and operation temperature must be still improved for specific applications. Therefore, scientists are interested in hetero-structured metal oxides such as TiO_2_, to increase their sensing performances. The sensing performances of heterojunction enhance in virtue of band alteration at the interface between different materials. This provides charge transfer through the interface from one material to another by creating charge-space region and adsorption sites [17, 28-32]. WO_3_ is one of the best candidates for heterostructure due to its highly reactive nature against various VOCs [33,34]. WO_3_/TiO_2_ heterostructures have been widely investigated for photocatalytic, thermochromic and supercapacitor applications [35-37]. However, investigation of gas sensing properties of WO_3_/TiO_2_ heterostructures is still limited. L.E. Depero et al. [38] and D-S. Lee et al. [39] fabricated TiO_2_-WO_3_ sensors with sputtering and coprecipitation methods, respectively, and explored their NO3 sensing performances. Y. Yao et al. [40] fabricated TiO_2_-WO_3_ composite coatings and explained the enhanced methane sensing properties by creation of heterojunctions between the TiO_2_ and WO_3_ interface. S.M. Zanetti et al. investigated the WO_3_ doping effect on TiO_2_ nanocrystalline powders and estimated their excellent humidity sensing properties [41]. W. Meng et al. synthesised TiO_2_ powder-core WO_3_ shell composite sensing electrode and demonstrated a better NH3 sensing performance [42]. Even though, MOX heterostructures have potential applications in chemical gas sensors [43-45], there are limited study on the sensing properties of WO_3_/TiO_2_ NRs heterostructures. Especially, studies with various WO_3_ loading are considerably poor. 

In this study, WO_3_/TiO_2_ NRs with various WO_3_ loading were obtained for chemical gas sensors. The enhancing sensor performance of SMO gas sensors with decorating of highly ordered 1D n-type TiO_2_ NRs by n-type WO_3_ is the motivation of this study. Highly ordered TiO_2_ NRs were synthesised by hydrothermal method on TiO_2_ seed-layer coated FTO substrates. Then, WO_3_ layers were coated on TiO_2_ NRs by magnetron sputtering technique with different thicknesses. Structural and morphological characterisation of WO_3_/TiO_2_ heterostructures were investigated. Gas sensor performances of heterostructures were studied against ethanol, toluene, xylene, acetone and humidity at various temperatures.

## 2. Materials and methods

FTO substrates (8 Ω/sq) was obtained from Sigma-Aldrich (ChemieGmbH, Hamburg, Germany). Hydrochloric acid (HCl, 37%) was acquired from Sigma-Aldrich. Titanium (IV) isopropoxide (TTIP, 97 +%) and sodium tungsten oxide dihydrate (Na_2_WO_3_ ·2H_2_O, 95%, crystalline) were provided from Alfa Aesar (city, country?). Titanium target (Ti, 99.995% purity and 2.00” diameter × 1.25” thick) and tungsten target (W, 99.95% purity and 2.00” diameter × 1.25” thick) were purchased from Kurt J. Lesker Company (city, country?). Distilled (DI) water (18 MΩ) was utilised through the all experiments.

### 2.1. Fabrication of WO_3_ modified TiO_2_ NRs heterostructures

The WO_3_/TiO_2_ heterostructures were obtained on the basis of different studies in the literature. A compact TiO_2_ seed-layer with a thickness of approximately 50 nm was deposited on the FTO substrate to prevent shorting before the growth of TiO_2_ NRs as reported previously in literature [46]. First, FTO substrates were purified by acetone, isopropanol and DI water in ultrasonic bath for 10 min, respectively. Then, Ti seed-layer was deposited by RF magnetron sputtering on the FTO substrates. Sputtering of Ti thin film (TF) process was performed in 5m Torr Ar atmosphere, with applied power of 100 W for 25 min. Finally, samples were annealed in the air atmosphere at 500 ˚C for 3 h. During the thermal oxidation process, Ti layer reacts with oxygen molecules in the air and transforms to TiO_2_ layer [47].

TiO_2_ NRs were synthesised by hydrothermal method on TiO_2_ seed-layer coated FTO substrates. Firstly, 40 mL DI water and 40 ml HCl was mixed. Then, 0.9 ml TTIP was added drop by drop on previously mixed solution. Finally, the resulting mixture was stirred for 1 h at room temperature to obtain a homogeneous solution. This precursor solution was poured into a 250 mL autoclave, and TiO_2_ seed-layer coated FTO substrates were placed vertically into the autoclave. Then the autoclave was sealed, placed into the temperature-controlled oven and thermally treated at 170 °C for 15 h [22, 48]. After thermal treatment, the samples were removed from autoclave, rinsed in DI water and dried under dry air flow.

**Figure 1:**
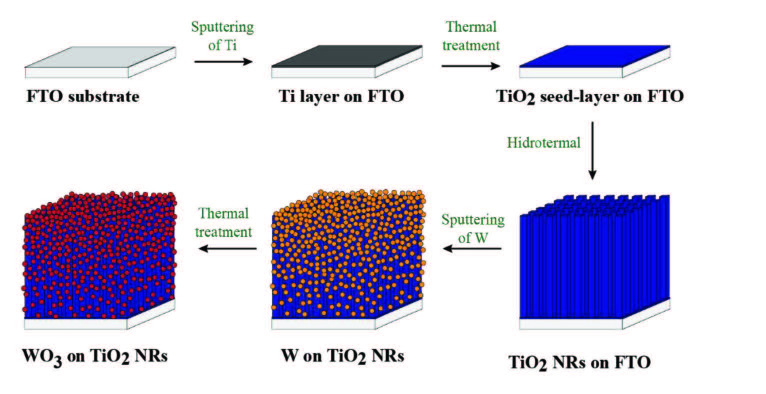
Schematic illustration of WO_3_/TiO_2_ NRs fabrication process.

WO_3_ layer was deposited on TiO_2_ NRs by the thermal oxidation method. Firstly, W layer was deposited by RF magnetron sputtering. Deposition of W layer was performed at 5 m Torr with RF power of 120 W for 1, 2, and 3 min under Ar atmosphere. Then, deposited W layer was thermally oxidised at 450 °C for 1 h to obtain the WO_3_ layer [49]. Sensor fabrication process was illustrated in Figure 1. Pristine TiO_2_ NRs sample was named as TiO_2_, and WO_3_-modified TiO_2_ NRs samples were named as WT-1, WT-2 and WT-3 in accordance with W deposition time of 1, 2, and 3 min, respectively.

The morphological and structural characterisations of fabricated samples were performed by scanning electron microscopy (SEM) equipped with energy dispersive X-ray spectroscopy (EDX) (Philips XL 30S) and X-ray diffraction (XRD) (Rigaku D-max, RINT-2200 series, X-ray diffractometer with Cu-Kα radiation, λ = 0.15418 nm), respectively.

### 2.2. Gas sensing measurements 

**Figure 2:**
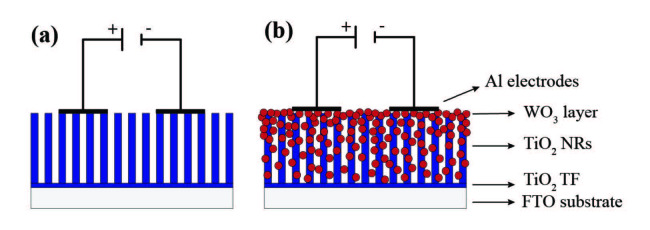
Schematic illustration of a) pristine TiO_2_ NRs and b) WO_3_ decorated TiO_2_ heterostructure sensor devices.

WO_3_-decorated TiO_2_ NRs were examined for VOCs and humidity sensing performance. To perform electrical measurements, aluminium contact electrodes (thickness 200 nm) were evaporated on the samples with Leybold Univex 450 (city, country?) thermal evaporation system. Schematic illustration of sensor fabrication was given in Figures 2a and 2b. The sensors were placed into a test chamber with 1 L in volume. A high purity dry airline was connected to the test chamber. The dry air flow and the concentration of gases were controlled by flow meters and a multi gas controller – MKS 647C. Working temperature of devices was controlled by a Lakeshore 340 (city, country?). Keithley 6517A electrometer (city, country?) was used for current vs. time characteristics during gas sensing measurements. The atmosphere in the test chamber was cleaned by dry air flow. When the electric current reached a steady value, VOCs was sent to the test chamber. Humidity sensing performances of the sensors were also characterised. VOCs were generated by bubbling method [25]. Antoine’s equation was used to calculate VOCs’ concentration. All data were reported as a sensor response defined as follows [50];

S_R_ = ∆I⁄I_0_,

where ∆I is the change in the current value when sensors were exposed to target gas molecules, I0 is the baseline current value measured under dry air flow condition. Response (t90_res_) and recovery (t90_rec_) times are defined as the time required the sensor to achieve 90% ∆I of its current form [51,52].

## 3. Results and discussion

### 3.1. Material characterisation 

**Figure 3:**
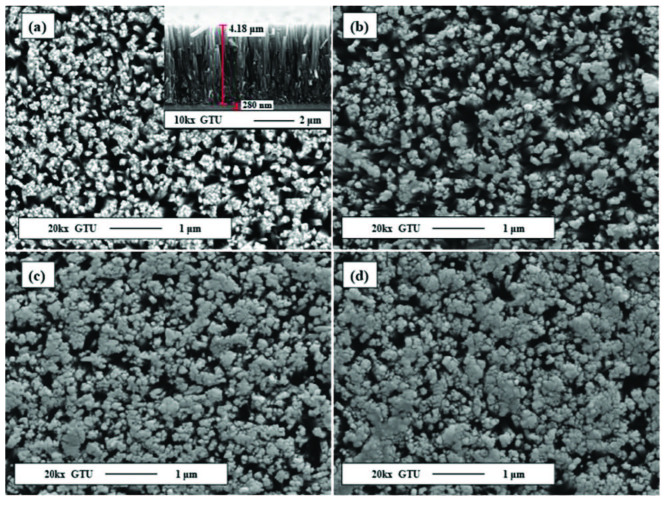
SEM images of a) TiO_2_ NRs, b) WT-1, c) WT-2, and d) WT-3.

The surface morphology and elemental analysis were performed by SEM and EDX, respectively. The SEM images indicate that TiO_2_ NRs are vertically aligned and homogenously covered on the substrate surface as seen in Figure 3a. The NRs were approximately 100 nm in diameter and 4.18 µm in length (Figure 3a inset). Figures 3b-3d show increasing amount of WO_3_ layer on the TiO_2_ NRs. The amount of WO_3_ depends on W sputtering time.

**Figure 4:**
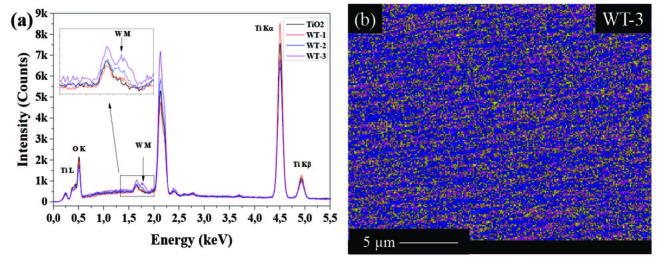
a) EDX spectrum of TiO_2_, WT-1, WT-2, and WT-3 samples and b) EDX mapping of WT-3 sample. In the mapping image, O, Ti, and W were representing yellow, blue, and purple, respectively.

Figure 4 shows the presence of the titanium, tungsten and oxygen in the rods. EDX spectrum of the samples in Figure 4a shows that tungsten is not present in TiO_2_ sample and atomic distribution of W are 0.5%, 0.94% and 1.11% in WT-1, WT-2 and WT-3 samples, respectively. The amount of W particles is correlated with sputtering time of W. The EDX mapping of WT-3 is given in Figure 4b. W particles homogeneously covered the surface of TiO_2_ NRs as seen in Figure 4b.

**Figure 5:**
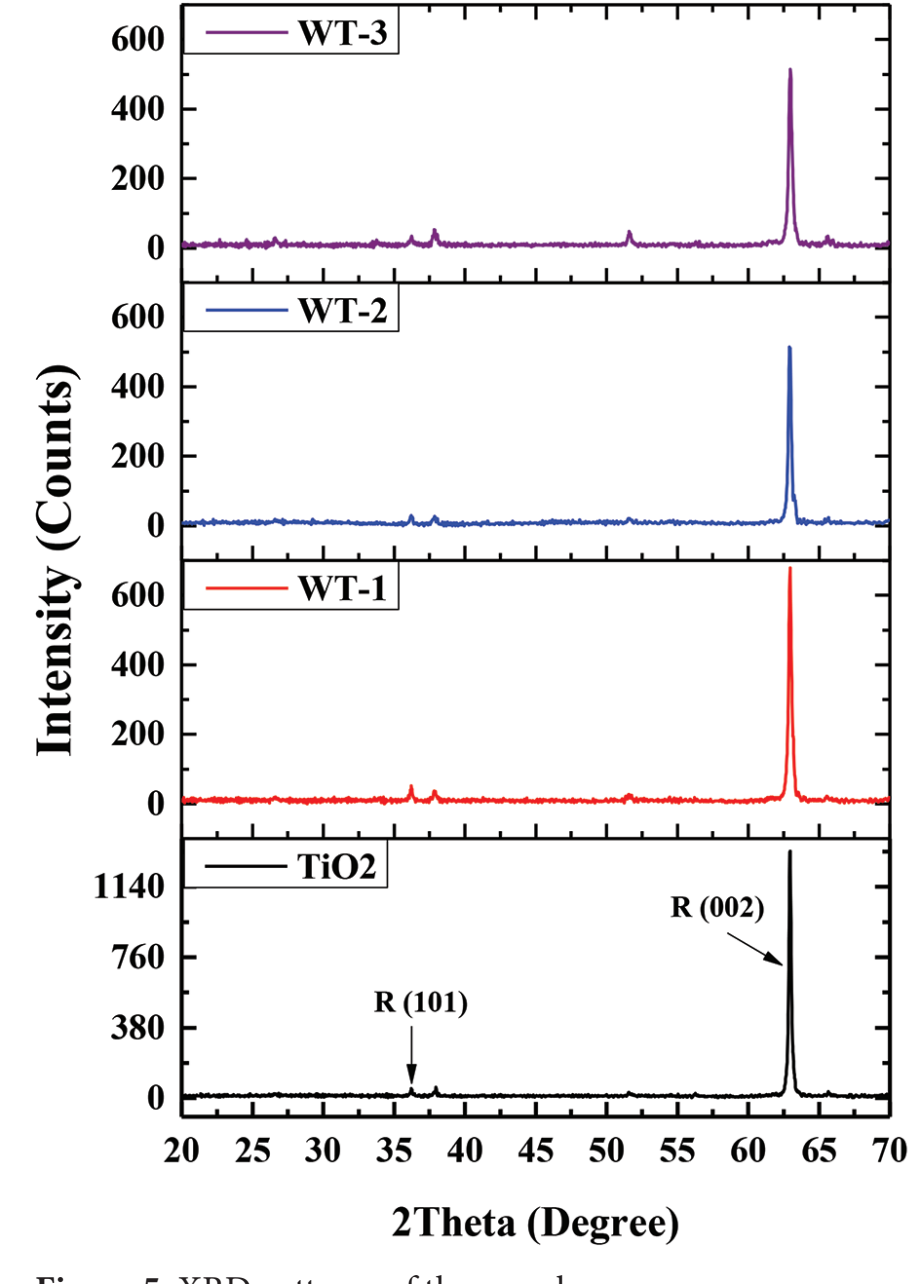
XRD patterns of the samples.

**Figure 6:**
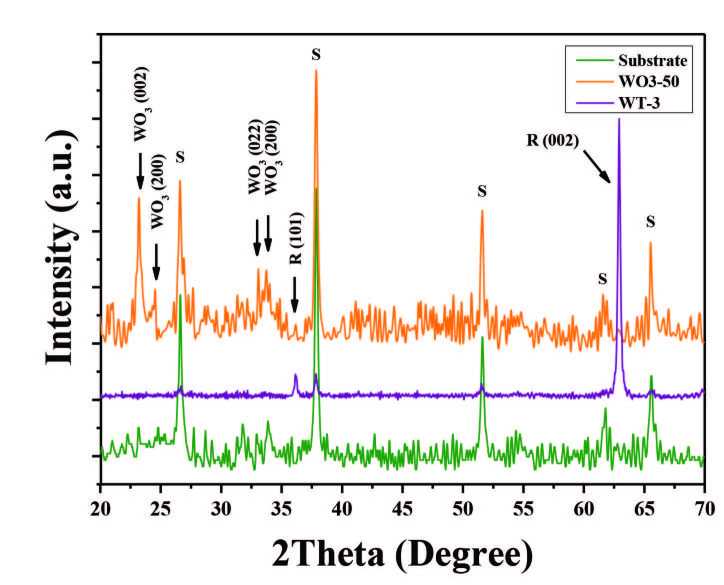
Comparison of XRD patterns of FTO substrate, 50 nm thick WO3 thin film, and WT-3 sample.

XRD patterns of the samples are given in Figure 5. According to the XRD patterns, the diffraction peaks 36.1˚ and 62.8˚ were attributed to (101) and (002) crystal planes of rutile TiO_2_, respectively (PDF card number 00-021-1276). The intensity of the diffraction peaks 36.1˚ and 62.8˚, which refer to rutile TiO_2_, decrease in samples WT-1, WT-2 and WT-3. This can be explained by decorating of TiO_2_ NRs with WO_3_ [53]. In addition, there are no observed diffraction peaks related with WO_3_ in the XRD pattern, which might be due to poor signal formation from small amount of material loading. In order to identify the WO_3_ XRD pattern, WO_3_ TF with a thickness of 50 nm (WO_3_-50) was deposited by RF magnetron sputter system, and subsequently thermal oxidised on FTO substrate. In Figure 6 comparative XRD results are shown for WO_3_-50, WT-3 and pristine FTO substrate. Diffraction peaks at 2θ = 23.2⁰, 24.54⁰, 33.1⁰ and 34⁰ can be assigned to monoclinic WO_3_ (002), (200), (022) and (220) reflections, respectively (PDF card number 00-043-1035). The peaks which marked as “S” belong to FTO substrate (PDF card number 00-046-1088).

Growth of TiO_2_ NRs can be explained with two continuous reactions;

Ti(OR)4+H2O→Ti(OH)4+4ROH(hydrolysis)

Ti(OH)4→TiO2,xh2O+(2-x)H2O(condensation)

where R is ethyl, i-propyl, n-butyl, etc. [54].

Firstly, hydrolysis occurs by reaction of TiO_2_ precursor (TTIP) with water and TTIP transforms to titanium alkoxide. Then, titanium alkoxide forms a complex with water. The acidic platform controls the rate of complex formation. Finally, the high temperature and pressure condition accelerates hydrolysis process and appeared complex starts to deposit onto the substrate as TiO_2_ NRs in rutile phase [55]. The fundamental reason of growing highly ordered NRs in the deposition process of titanium complexes onto the substrate is surface energy. In the TiO_2_ phase, the lowest surface energy has (110) face. It means that [001] direction, parallel to (110) plane, is the theoretically preferable growth direction. The powerful (002) pick in XRD pattern is the proof of the growth of the highly aligned TiO_2_ NRs along [001] direction [56].

### 3.2. Gas sensing properties

**Figure 7:**
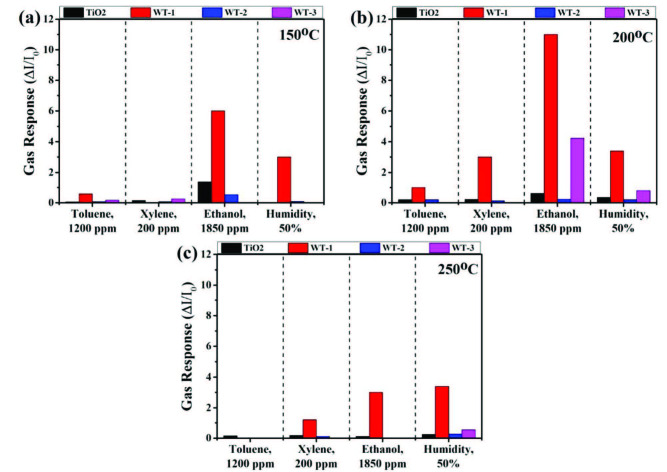
Sensor responses of samples at constant concentration against all tested gases at a) 150 °C, b) 200 °C, and c) 250 °C.

Gas sensor measurements of fabricated sensors were performed under toluene, xylene, acetone, ethanol, and relative humidity ambient in an operation temperature range between 100 °C and 250 °C. There was no observed sensor response signal from all the samples against any gases at 100 °C. Also, pristine TiO_2_ and WO_3_/TiO_2_ heterostructures could not sense acetone molecules for all operation temperatures. Operation temperature dependent sensor response results of all sensors are given in Figure 7 with bar diagrams.

**Figure 8:**
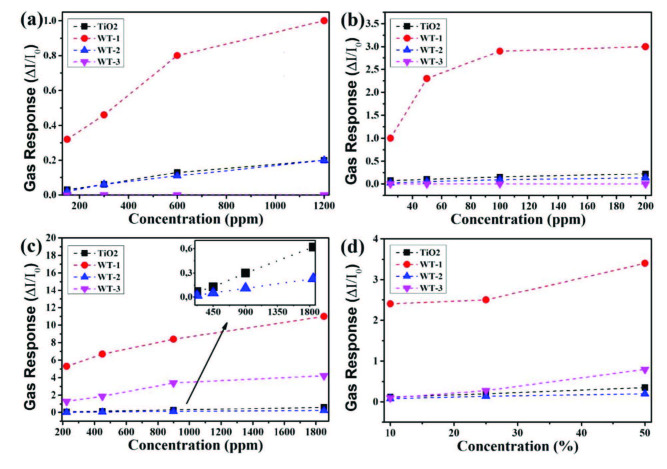
Gas concentration dependence of sensor response against a) toluene, b) xylene, c) ethanol, and d) relative humidity at 200 °C.

After all sensor measurements at different operation temperatures, 200 °C is identified as the optimal operation temperature for all sensors due to the highest sensor response values against each gas. At the optimal operation temperature, WT-1 sensor showed an excellent sensing performance against 1850 ppm ethanol as seen in Figure 7b. Sensor performance toward ethanol is drastically increased with the effect of WO_3_ on the surface. Sensor response of WT-1 is 18-fold higher compared to pristine TiO_2_ NRs at 200 °C. WO_3_ plays a key role on the surface as catalyst and increases the sensor response. Also, sensor response values of WT-1 are highest against all tested gases due to catalytic effect of WO_3_. After the identifying optimal operation temperature, concentration dependence of sensor response was investigated at 200 °C. Concentration dependence of sensor response is given in Figure 8. 

The sensing performances of aromatic compounds such as toluene and xylene change by increasing amount of loaded WO_3_-layer on the TiO_2_ NRs as seen in Figures 8a and 8b. Sensing performances of pristine TiO_2_ NRs are similar toward 1200 ppm toluene (sensor response = 0.2) and 200 ppm xylene (sensor response = 0.22) and decrease linearly by decreasing concentration of the aromatic compounds. The best sensing responses of aromatic compounds were shown by WT-1 sensor. Moreover, WT-1 sensor has saturated by increasing of concentration as clearly seen in xylene sensing performance. At low concentrations, the sensor response increases rapidly against xylene and changes slowly by increasing concentration after 100 ppm. The sensor response against aromatic compounds of WT-2 less than WT-1. Moreover, WT-3 sensor could not sense toluene and xylene. Fabricated sensors show good linear characteristics in a certain concentration range of ethanol as seen in Figure 8c. Sensor response value increases with the increment of ethanol concentration. Figure 8d shows sensor response of samples against different relative humidity concentrations at 200 °C. WT-1 sample exhibited the highest sensor response at every concentration of relative humidity.

**Figure 9:**
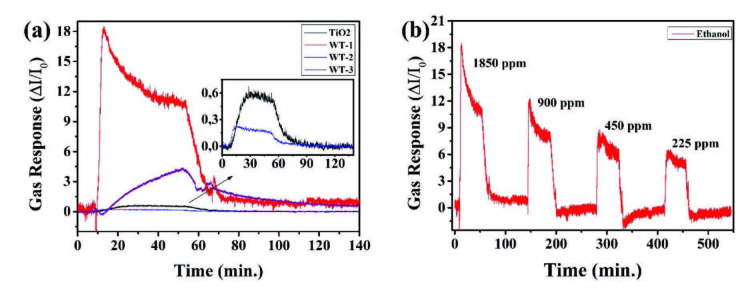
a) Sensor response of 1850 ppm ethanol for all samples and b) different ethanol concentrations for WT-1 sample at 200 °C.

The investigation of concentration dependence of sensor response demonstrate that all sensors are most sensitive against ethanol molecules at optimal operation temperature. Sensor response of all samples against 1850 ppm ethanol and different ethanol concentrations for WT-1 sample at 200 °C are given in Figure 9. 

It’s clear that the signal returns to baseline after turning off the ethanol and purging with dry air. WO_3_ modified TiO_2_ NRs heterostructures showed enhanced sensor properties compared to the pristine TiO_2_ NRs sensor. Sensing mechanisms of MOX sensors that are composed of only one type of materials have been studied and well explained in the literature [57,58]. Enhanced sensor properties can be attributed to the catalytic effect of WO_3_. In this case, WO_3_ plays a role as a catalyst material in the reaction between analyte gas and TiO_2_. If the surface coverage of the WO_3_ increase, the catalytic role of the WO_3_ turns into a sensing layer, so a lower sensor performance generally is observed [59]. In Figure 3, SEM data also clarifies more coverage on the surface for WT-2 and WT-3. Previous works have also reported the enhanced sensor properties due to the catalyst role in heterostructures [17, 60-62]. WT-1 sample exhibited enhanced ethanol response than others and its concentration dependence sensor response performance was illustrated separately in Figure 9b. During the exposure, the response increased rapidly, then the rate of increment stopped and slightly declined to reach the saturation. While purging the sensor, the response decreases rapidly and reaches baseline. Response times (t90_res_) of WT-1 sensor are 6 min for each ethanol concentrations. The sensor showed a very stable sensing characteristic against ethanol. On the other hand, recovery times (t90_rec_) of WT-1 sensor are 15, 14, 8, and 6 min for 1850, 900, 450, and 225 ppm ethanol, respectively. These time values are better than the ones in our previous ethanol sensor studies [22,50].

**Figure 10:**
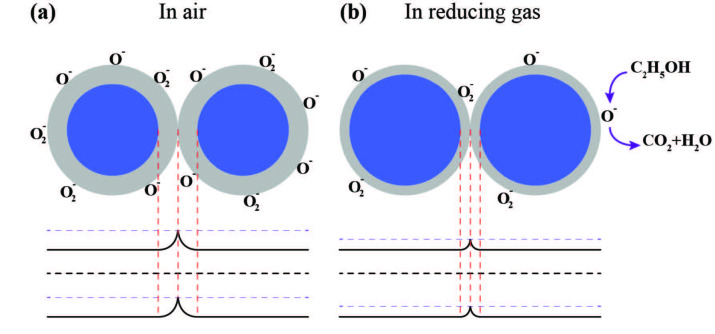
The schematic illustration of n-type metal oxide gas sensors grain boundary when exposed to a) air ambient and b) reducing gas ambient.

SMO materials such as TiO_2_ and WO_3_ have oxygen deficiencies on crystalline surface due to their specific stoichiometry. As a result of this condition, free electrons appear in the conduction band of SMO material. Therefore, this type of semiconductors is named as n-type semiconductor. Gas sensing mechanism of n-type SMO materials generally can be explained with oxygen adsorption on the surface as given in Figure 10. When the n-type SMO was exposed to ambient, oxygen molecules in the air would be adsorbed on the surface of SMO with capturing by charge carriers (free electrons) (Figure 10a). Decrease in number of charge carriers leads to appearance of depletion layer between the grain boundaries that limits the electron transfer. Therefore, the depletion layer width and contact barrier height between two adjacent grains will be increased by the remaining number of oxygen molecules. The higher contact barrier leads to lower conductance of n-type SMO. When n-type SMO material is exposed to reducing gas molecules, these molecules react with the preadsorbed oxygen molecules (Figure 10b). Then, the depletion layer width and contact barrier height between two adjacent grains decreases again. As a result, this reaction leads to increasing of n-type SMO materials conductance [63].

**Figure 11:**
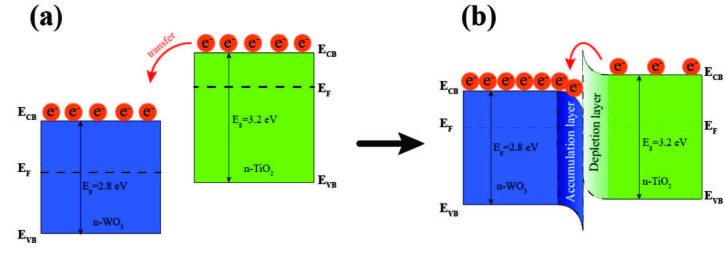
Energy band diagram of a) n-type TiO2 and n-type WO3 with different Fermi levels and direction of electrons migration to reach thermal equilibrium; b) formation of n-WO3/n-TiO2 heterostructure with accumulation and depletion layer.

When there is contact formed between TiO_2_ and WO_3_, these two different n-type SMO materials behave as a new sensing material named as n-WO_3_/n-TiO_2_ heterostructure, as shown in Figure 11. Because Fermi levels of TiO_2_ is higher than that of WO_3_, the electrons are transferred from conduction band of TiO_2_ to the conduction band of WO_3_ (Figure 11a). This process would continue until equalising of the Fermi level between WO_3_ and TiO_2_ occurs. The formation of n-n-type heterostructure leads to the creation of an electron depletion layer in TiO_2_ and an electron accumulation layer in WO_3_ (Figure 11b). The accumulation layer of WO_3_ would be enhanced oxygen adsorption in air ambient [40,48-50,64].

## 4. Conclusion

WO_3_/TiO_2_ heterostructures were fabricated to investigate VOCs sensing performance. According to morphological characterisation, WO_3_ uniformly covered the entire highly ordered TiO_2_ NRs surface. XRD investigation shows that TiO_2_ NRs was grown on rutile phase and was highly aligned along the [001] direction. XRD peaks of WO_3_ on samples did not exist due to their small amounts. However, further investigation illustrated that WO_3_ will grow in monoclinic phase with the thermal oxidation method. TiO_2_ and WO_3_/TiO_2_ heterostructures were tested against VOCs such as toluene, xylene, acetone and ethanol, and relative humidity. It was observed that n-n-type WO_3_/TiO_2_ heterostructure advanced the sensor performance of TiO_2_ NRs against almost all tested gases, except acetone, which is not detected with any sensors. WT-1 sensor showed the best sensor performance compared to TiO_2_, WT-2 and WT-3 sensors. Ethanol sensing response of WT-1 sensor was 18-fold higher than pristine TiO_2_ NRs at 200 °C. The enhanced gas sensor performance of WO_3_/TiO_2_ heterostructure is attributed to n-n type heterostructure formation that leads to the formation of depletion and accumulation layers. According to our findings, n-WO_3_/n-TiO_2_ heterostructures have a high potential for ethanol sensor applications.
